# Perceived Likelihood of Developing Diabetes Among High-Risk Oregonians

**Published:** 2005-10-15

**Authors:** Angela M Kemple, Amy I Zlot, Richard F Leman

**Affiliations:** Cardiovascular, Diabetes, Nutrition, and Physical Activity Section, Washington State Department of Health; At the time of the study, Ms. Kemple was a Research Analyst with the Oregon Department of Human Services, Diabetes Prevention and Control Program, Portland, Ore; Genetics Program, Oregon Department of Human Services, Portland, Ore; Health Promotion and Chronic Disease Prevention Program, Oregon Department of Human Services, Portland, Ore

## Abstract

**Introduction:**

Prevention of diabetes in people at highest risk for developing the disease is an important public health opportunity, considering the disease's increasing prevalence, its devastating impact on health and its high economic cost, the availability of efficacious and cost-effective treatments to reduce complications, and recent evidence that it can be delayed or prevented with lifestyle interventions.

**Methods:**

The Oregon Diabetes Prevention and Control Program collected and analyzed responses from a statewide telephone survey conducted in 2003 to determine whether Oregon adults at highest risk for diabetes 1) believed that they were at risk for developing diabetes in the future, 2) had talked with a health care professional about diabetes, and 3) had been tested for the disease. Pearson chi-square tests and logistic regression analyses were conducted to identify independent associations of select characteristics with the study factors of interest.

**Results:**

Even among respondents at highest risk for developing diabetes, at most one third reported being concerned about developing diabetes, one fifth reported having discussed their risk with a health professional in the previous year, and less than half reported having been tested for diabetes by a health provider in the previous year. After adjusting for multiple factors, we found that having a family history of diabetes was consistently associated with perceived risk of developing diabetes, discussion about diabetes with a health professional, and diabetes testing.

**Conclusion:**

Many Oregon adults at high risk for developing diabetes are unconcerned about their risk for developing the disease, and few have discussed their risk of diabetes with a health professional. Findings from this study suggest the need for increased recognition of future diabetes risk by high-risk individuals and health professionals to help translate diabetes prevention into practice.

## Introduction

Diabetes is a growing public health problem. Nationally, the prevalence of diabetes increased almost 50% during the previous decade (1). In Oregon, the percentage of adults who reported having been told by a doctor that they had diabetes increased from 4% in 1995 to 6% in 2003 (2). Diabetes is associated with morbidity and mortality; it is a leading cause of death and is associated with new cases of end-stage renal disease, lower limb amputations, blindness, and cardiovascular disease (3). It is a chronic, progressive, degenerative disease that has devastating effects on quality of life and results in high costs for individuals and society because of its complications, hospitalizations, and lost productivity (3). When the disease is diagnosed, diabetes complications can be reduced through evidence-based, cost-effective treatment strategies, but often these treatments are underused (3).

Additional increases in diabetes prevalence are likely in light of projected changes in the age and racial and ethnic composition of the U.S. population, overall population growth, and increasing numbers of people who are overweight, obese, or less physically active (3,4). Fortunately, the recent success of major diabetes prevention trials demonstrates that development of type 2 diabetes can be delayed and in some cases prevented in high-risk individuals through lifestyle modifications such as modest weight reduction and regular physical activity (5,6).

Risk factors for type 2 diabetes are well established and include older age, obesity, family history of diabetes, prior history of gestational diabetes, history of bearing an infant weighing 9 lb or more at birth, physical inactivity, and prediabetes (a condition in which blood glucose levels are elevated, although not enough to meet the diagnostic criteria for diabetes) (7). In addition, type 2 diabetes is more common among African Americans, Hispanic and Latino Americans, American Indians, and some Asian Americans and Pacific Islanders than among non-Hispanic whites (7).

More recently, recognition of populations at higher risk for developing prediabetes has been increasing. People who are both overweight (body mass index [BMI] ≥25.0 kg/m^2^) and aged 45 years and older are at particularly high risk (8). Younger overweight individuals who have additional risk factors for type 2 diabetes are also at increased risk (8). It is estimated that almost one fourth of overweight adults aged 45 to 74 years — 12 million nationwide — have prediabetes (9). Based on estimates from the Behavioral Risk Factor Surveillance System (BRFSS) surveys, 673,000 Oregonians are 45 years and older and overweight. As many as 152,000 of these individuals may have prediabetes and could benefit from interventions to help them avoid developing type 2 diabetes (10).

Prevention of diabetes in high-risk people is an important opportunity for public health professionals. Diabetes prevalence is increasing because of contemporary lifestyle changes (1,3,4), and the disease shortens life expectancy and has devastating effects on quality of life (3). Effective and economical treatment strategies exist to reduce complications in people who already have been diagnosed with diabetes (3), and recent evidence shows that type 2 diabetes can be delayed or prevented with lifestyle interventions that have ancillary benefits (5,6). Increasing awareness of primary prevention strategies in people at highest risk for diabetes and effectively promoting prevention interventions in medical and community settings will be a challenge. More information is needed about perceptions of diabetes risk and prevention in high-risk individuals and among health care professionals.

In this study, the Oregon Diabetes Prevention and Control Program collected and analyzed responses from a statewide telephone survey conducted in 2003 to determine whether Oregon adults at highest risk for diabetes 1) believed that they were at risk for developing diabetes in the future, 2) had talked with a health care professional about diabetes, and 3) had been tested for the disease.

## Methods

Information on Oregon adults' perceptions of diabetes risk and prevention was collected from Oregon's 2003 BRFSS, a state-based, random-digit–dialed household telephone survey. A disproportionate stratified sample design was used to obtain a probability sample of the noninstitutionalized, adult population aged 18 years and older in Oregon (11).

### Survey measures

Initially, survey respondents were asked whether a doctor had ever told them they had diabetes. Respondents considered not to have diabetes (which included women who were told they had diabetes only during pregnancy) were asked a series of questions about diabetes risk factors, their perceived risk of developing diabetes, and any diabetes-related discussions or testing that had occurred in the health care setting (Table 1).

Respondents were also asked about their extent of participation in moderate or vigorous activity in a usual week. Information about physical activity was collected using the standard BRFSS physical activity core module (12). Respondents were categorized by physical activity levels as follows: 1) met Centers for Disease Control and Prevention (CDC) recommendations (either moderate-intensity activity during leisure time for 30 minutes or more on 5 or more days per week or vigorous physical activity during leisure time for 20 minutes or more on 3 or more days per week; 2) insufficient activity (some physical activity but not enough to meet CDC recommendations); or 3) inactive (less than 10 minutes of moderate-intensity physical activity during leisure time in a usual week). BMI was calculated based on self-reported height and weight and was categorized as follows: 1) healthy weight (BMI <25.0 kg/m^2^), 2) overweight (BMI 25.0–29.9 kg/m^2^), or obese (BMI ≥30.0 kg/m^2^).

High-risk groups assessed included people with a family history of diabetes, people who were overweight or obese, people who were physically inactive, people who were aged 45 years and older, and people of Hispanic or Latino ethnicity. Data for racial and ethnic populations other than non-Hispanic whites or Hispanics and Latinos were combined because when analyzed separately, the sample was too small for meaningful analysis. Respondents also indicated whether a doctor, a nurse, or another health professional had ever told them they had high blood pressure or high cholesterol.

We assessed separately the group that was overweight and aged 45 years and older because this group is at particularly high risk for prediabetes, and diabetes testing for people in this group is highly recommended (8). The total number of risk factors commonly associated with diabetes was also determined for each respondent without diabetes (including ages 45 years and older, obesity, a family history of diabetes, and inactivity). Education level and annual household income were also specified for each respondent. Table 2 shows the risk factor categories and select characteristics.

Three survey questions were used to assess the relationship between the primary outcomes of interest (diabetes risk perception, diabetes discussions with a health care professional, and diabetes testing) and access to medical care. Respondents were asked to answer yes or no to the following questions:

1. Do you have any kind of health care coverage, including health insurance, prepaid plans such as HMOs, or government plans such as Medicare?
2. Do you have one person you think of as your personal doctor or health care provider?
3. Was there a time during the last 12 months when you needed to see a doctor but could not because of the cost?

### Data analysis

Before analyzing the data, we weighted the sample responses to adjust for differences in probability of selection and nonresponse and to derive estimates that more accurately reflect the population from which the sample was drawn (i.e., adult Oregonians as of July 1, 2003) (13). Pearson chi-square tests were used to explore associations among perceived risk for diabetes, discussion of diabetes with health care professionals, diabetes testing, and the presence or absence of diabetes risk factors. Logistic regression analysis was used to assess significant univariate factors to determine the independent effect of important risk factors on each of the outcomes. Respondents who reported "don't know" or refused to answer questions were excluded from analysis. Percentages, odds ratios (ORs), and 95% confidence intervals (CIs) were calculated using the survey analysis procedures in STATA software, version 7 (StataCorp LP, College Station, Tex). The Taylor series linearization method was used to compute the variance of survey estimates that were appropriate for the complex sample design.

## Results

Based on the Council of American Survey Research Organizations response rate formula, the proportion of all eligible respondents in the sample for whom an interview was completed was 50% (14). A total of 1974 respondents completed telephone interviews, and 1810 (92.7%) reported that they had not been diagnosed with diabetes; 21 (1.1%) women reported that they had been diagnosed only during pregnancy, so these women were considered not to have diabetes. The remaining 141 adults (6.2%) who reported having been told by a doctor that they had diabetes and 2 adults with unknown diabetes status were excluded from additional analyses.

Among respondents without known diabetes, 51.3% were women, and the mean age was 45 years (range 18 to 99 years). The majority (84.7%) reported being non-Hispanic white; 30.4% had completed high school but did not go on to college, and 59.6% had some college education. Household earnings assessments revealed that 31.6% had an annual household income of $25,000 to $49,999, and 35.3% earned $50,000 or more. Respondent access to medical care was as follows: 80.4% had some form of health care coverage, 75.1% had at least one person they thought of as their personal doctor or health care provider, and 14.5% reported they were unable to seek medical care at some time in the previous 12 months because of cost.

The distribution of selected risk factors for diabetes and prediabetes was as follows: 27.5% had a family history of diabetes, 37.7% were overweight, 20.6% were obese, 36.9% were insufficiently active during leisure time, and 10.7% were inactive during leisure time. Comorbidities that increase the risk of diabetes complications were common: 20.5% had been told by a doctor, a nurse, or another health professional that they had high blood pressure; 33.5% reported being told by a doctor, a nurse, or another health professional that they had high cholesterol; and 21.7% were current smokers. When four common risk factors (ages 45 years and older, obesity, a family history of diabetes, and inactivity) were analyzed together, 32.1% had none of these risk factors, 38.3% had one, 21.7% had two, and 7.9% had three or four. In addition, 19.8% were aged 45 years and older and overweight.

Overall, only 14.5% of respondents were at least somewhat or very worried about developing diabetes in the next 10 years, 11.4% had talked about diabetes with a health care professional in the previous year, and 25.6% had been tested for diabetes by a health care provider in the previous year (Table 2). Significant associations were found among all three factors of interest (Table 1).

### Perceived risk of developing diabetes

Results from the bivariate analysis (Table 3) show that the likelihood of being concerned about developing diabetes was higher among respondents who were women, were Hispanic or Latino, were obese, were insufficiently active or physically inactive, had not been able to see a doctor at some time in the previous 12 months because of cost, and had a family history of diabetes. Respondents aged 65 years and older and those with more than a high school education were less likely to be worried. Respondents with two or more risk factors for diabetes were more likely than those with fewer risk factors to be worried about developing diabetes in the future. In no group did more than 34% of respondents express concern about their risk of developing diabetes in the future.

After including all significant variables in a single logistic regression model, a family history of diabetes (OR 4.7 [95% CI, 3.3–6.7]), obesity (OR 2.8 [95% CI, 1.8–4.4]), being Hispanic or Latino (OR 2.6 [95% CI, 1.3–5.2]), being insufficiently active (OR 1.6 [95% CI, 1.1–2.3]), and being a woman (OR 1.6 [95% CI, 1.1–2.3]) were all independently associated with concern about developing diabetes in the next 10 years. Respondents aged 65 years and older were less likely to be worried about developing diabetes in the future than those aged 18 to 44 years (OR 0.2 [95% CI, 0.1–0.4]).

### Diabetes discussion with a health care professional

Bivariate analyses (Table 4) indicate that the likelihood of talking with a health care professional in the previous year about diabetes was higher among respondents who were women, had a family history of diabetes, were obese, had a history of high blood pressure, and had a personal health care provider. The likelihood of discussing diabetes with a health care professional also increased with increasing number of risk factors for diabetes. After adjusting for multiple factors, a family history of diabetes (OR 2.9 [95% CI, 2.0–4.1]), being a woman (OR 1.8 [95% CI, 1.2–2.6]), and obesity (OR 1.6 [95% CI, 1.1–2.5]) were still independently associated with a history of talking with a health care professional in the previous year about diabetes.

### Diabetes testing

Findings from bivariate analyses (Table 5) show that the likelihood of being tested for diabetes by a health care provider in the previous year was higher among respondents who were women, were aged 45 years and older, were overweight or obese, had a history of high blood pressure or high cholesterol, had health care coverage, had a personal health care provider, and had a family history of diabetes. The likelihood of being tested increased with increasing number of risk factors for diabetes. Respondents who were aged 45 years and older and overweight were also more likely to have been tested for diabetes in the previous year than respondents who did not have this combination of risk factors for prediabetes. Three of the risk factors were independently associated with diabetes testing by a health care provider in the previous year: a family history of diabetes (OR 2.0 [95% CI, 1.5–2.8]), ages 65 years and older (OR 1.9 [95% CI, 1.3–2.8]), and a history of high blood pressure (OR 1.5 [95% CI, 1.1–2.1]).

## Discussion

To help translate primary diabetes prevention into practice, at-risk individuals and health care professionals must be aware of the risk factors for developing diabetes, talk to each other about diabetes, test for evidence of prediabetes, and begin preventive interventions. However, even among respondents in this study at highest risk for diabetes, at most one third reported being concerned about developing diabetes, one fifth reported having discussed their risk with a health professional in the previous year, and less than half reported having been tested for diabetes by a health provider in the previous year. Although our results show that respondents with more risk factors tended to be more aware of their risk for diabetes, fewer than one third of people at highest risk (i.e., those with three or four risk factors) were worried about developing diabetes in the future. These findings about risk perception are similar to findings from previous studies in the general population, which suggest that individuals tend to underestimate their risk for developing diabetes (15-17).

Discussing diabetes with a health care professional and testing for diabetes were also more likely among individuals with several diabetes risk factors. We are unsure whether these associations reflect more frequent health care visits because of the number of risk factors or result from respondents' risk perceptions. Although we were able to determine that adults who were worried about developing diabetes were more likely to talk with a health care professional and be tested for diabetes (Table 1), we were unable to determine the number of health care visits made.

Even though the American Diabetes Association recommends that fasting blood glucose or glucose tolerance testing should be considered for all individuals aged 45 years and older (8), our results show that respondents aged 45 to 64 years were no more likely to be worried about developing diabetes or to discuss diabetes risk with a health care professional than their younger counterparts. Respondents aged 65 and older were even less concerned. However, testing for diabetes did increase with increasing age.

Although obesity was consistently associated with increased perceived risk, being overweight was not independently associated. In addition, the respondents who were overweight and aged 45 years and older (a group at particularly high risk of developing prediabetes [8]) were no more likely to perceive being at risk for diabetes than younger respondents who were not overweight. Furthermore, this high-risk group was no more likely to report discussing diabetes risk with a health professional. In contrast, Harwell et al reported that among adults aged 45 years and older, being overweight was independently associated with perceived risk for developing diabetes and was also associated with having received medical advice regarding diabetes risk (16). In our study, the group of respondents that was overweight and aged 45 years and older was more likely to report having been tested for diabetes, a finding that is also different from that of another study by Harwell et al (18).

Even though older adults (aged 45 years and older) and the group that was overweight and aged 45 years and older were no more likely to be concerned about developing diabetes than their lower risk counterparts, the increased likelihood of testing among these high-risk groups may partly reflect health care providers' recognition that these adults are at higher risk for prediabetes and diabetes. Lower reported levels of perceived risk may also result from high-risk adults who have already been tested and not been diagnosed with diabetes.

Previous research has reported a twofold to sixfold higher risk of developing type 2 diabetes among individuals with a family history of diabetes compared with people who have no family history of diabetes (19). Although family history was strongly associated with all three study questions, among respondents with a family history the actual percentages of those who reported being worried about developing diabetes (31%), having talked with a health care professional (21%), and being tested for diabetes (38%) were still low. Pierce et al reported that family members of individuals with type 2 diabetes underestimate their own risk of developing diabetes (20). Another population-based survey of adults aged 45 years and older also noted that although perceived risk of developing diabetes was higher among respondents with a family history of diabetes, less than half actually considered themselves to be at risk (16).

Although Hispanic and Latino respondents were more worried about developing diabetes than non-Hispanic whites, they were no more likely to have talked with a health care professional about diabetes or to have been tested for diabetes. These results may be related to decreased access to medical care among Hispanics and Latinos. Additional analysis of Oregon's 2003 BRFSS revealed that Hispanic and Latino respondents were significantly less likely (48.1%) than non-Hispanic whites (84.4%) to have any kind of health care coverage or to have one person they thought of as their personal doctor or health care provider (47.5% vs 79.2%); they were significantly more likely (23.8%) than non-Hispanic whites (13.1%) to have had a time during the past 12 months when they were unable to seek medical care because of cost (A.M.K., unpublished data, 2005). Although the difference was not statistically significant, the low percentage of Hispanic and Latino respondents who had discussed diabetes risk with health care providers indicates the need for better access to medical care for these individuals and culturally appropriate education for health care professionals so that they will encourage diabetes discussions and testing.

The number of respondents was not sufficient to assess self-perceived risk of diabetes among racial and ethnic groups other than non-Hispanic whites and Hispanics and Latinos. Future research should explore diabetes perceptions and awareness of its prevention among other racial and ethnic populations that are at higher risk.

Discussing diabetes with a health care professional and diabetes testing were not found to be independently associated with access to medical care. We were unable to track the number of health care visits made to providers, which may have been a better indicator of medical care access and may have been associated with the major study factors of diabetes risk perception, diabetes discussions with health care providers, and diabetes testing. A previous population-based study on diabetes testing among adults aged 45 years and older found that a history of two or more visits to a health care provider in the previous year was independently associated with diabetes testing within the previous year (18).

### Limitations

All data were self-reported, which may have resulted in recall and nonresponse bias, especially for questions about diabetes testing, frequency and duration of physical activity, and weight and height used to compute BMI. Moreover, individuals who have diabetes but have not been diagnosed or do not remember being diagnosed may have been categorized as not having diabetes. The sample only represents individuals living in households with land-based telephones; individuals without telephones, those who used cellular phones exclusively, and those who were institutionalized were not represented (11).

We only asked respondents whether they had talked with a health care professional about diabetes or been tested for diabetes in the year preceding the survey date. Because of this restricted time frame, we were unable to obtain information about respondents who had been tested more than a year before the survey date, had received a negative result, and were not due for another test (8). If the time frame had been extended, the percentages of adults who had talked with a health care professional and been tested for diabetes may have been higher. In addition, among respondents who had been tested for diabetes, we were unable to determine the type of test performed (Table 1).

Several health behavior models describe the important impact of multiple health beliefs such as perceived severity, outcome expectations, self-efficacy, and perceived risk on an individual's likelihood of initiating a behavior change (21,22). In our study, BRFSS data were only collected on one health belief: a person's perceived risk for developing diabetes. Additional research is needed to determine whether Oregon adults at high risk for diabetes who are worried about developing diabetes actually believe that their risk is serious, believe the benefits of taking action outweigh the costs, believe they have the ability to change, and then actually make the necessary lifestyle changes to decrease their risk.

The cross-sectional nature of this study may have restricted our interpretation of certain findings. For example, certain high-risk respondents, such as older adults, may not have been worried about developing diabetes because they had already talked with a health care professional about their risk, been tested for the disease, and received a negative result. Prospective studies are needed to further elucidate the complex relationships among the primary outcomes of interest: perceptions of diabetes risk, discussions about diabetes with a health care professional, and testing for diabetes.

### Implications

Many Oregon adults at high risk for developing diabetes are unconcerned about their risk for developing the disease. Findings from our study suggest that high-risk individuals need to be more aware of their potential for developing diabetes, as do their health care professionals — an initial step toward translating diabetes prevention into practice. Effective public health messages about diabetes awareness could be incorporated into educational and screening interventions targeted toward populations at high risk for developing diabetes. These messages should address the risk of developing diabetes, the value of discussing diabetes risk with a health care professional, and ways to delay or even prevent the condition from developing with fairly simple lifestyle changes. Although health professionals are still designing targeted programs that identify individuals at increased risk of developing prediabetes or diabetes and offer appropriate education and screening strategies, findings from our study provide support for the potential benefits of such programs.

## Figures and Tables

**Table:** 

**Survey Question**	**Worried About Developing Diabetes in Next 10 Years^a^**		**Χ^2^_1_ * (P*)^c^**

	**Somewhat or Very Worried** ** (n = 249)** ** No. (%)^b^**	**Not at All or Slightly Worried** ** (n = 1528)** ** No. (%)^b^**	
Talked to health professional about diabetes in previous 12 months	67 (26.0)	145 (8.8)	51.9 (<.001)
Tested by health care provider for diabetes in previous 12 months	101 (36.7)	375 (23.6)	15.7 (<.001)

**Table:** 

**Survey Question **	**Talked to Health Professional About Diabetes in Previous 12 Months**		**Χ^2^_1_ * (P*)^c^ **

	**Yes** ** (n = 213)** ** No. (%)^b^**	**No** ** (n = 1560)** ** No. (%)^b^**	
Somewhat or very worried about developing diabetes in next 10 years	67 (33.3)	181 (12.1)	51.9 (<.001)
Tested by health care provider for diabetes in previous 12 months	157 (72.0)	317 (19.5)	192.3 (<.001)

**Table:** 

**Survey Question **	**Tested by a Health Care Provider for Diabetes in Previous 12 Months**		**Χ^2^_1_ * (P*)^c^ **

	**Yes** ** (n = 477)** ** No. (%)^b^**	**No** ** (n = 1244)** ** No. (%)^b^**	
Somewhat or very worried about developing diabetes in next 10 years	101 (21.1)	146 (12.5)	15.7 (<.001)
Talked to health professional about diabetes in previous 12 months	157 (32.2)	51 (4.3)	192.3 (<.001)

a Response categories were very worried, somewhat worried, slightly worried, or not at all worried.

b Percentages are weighted.

c *P* values <.05 indicate that diabetes risk perception, diabetes discussions with a health care professional, and diabetes testing are associated with each other based on the complex survey-design Pearson’s chi-square test. The design-based *F* statistic was used to calculate the chi-square value.

**Table 2:** Major Factors Used to Measure Risk Perception and Prevention Strategies, by Select Characteristics, Among Oregon Adults Without Diabetes, 2003 Oregon Behavioral Risk Factor Surveillance System

**Characteristics**	**Sample Size (N)**	**Worried About Developing Diabetes in Next 10 Years a,b**		**Talked About Diabetes With Health Care Professional in Previous Year^b^**		**Tested for Diabetes by Health Care Provider in Previous Year^b^**	

		**No. (%)**	***χ* ^2^_df _(*P*)^c^**	**No. (%)**	***χ* ^2^_df _(*P*)^c^**	**No. (%)**	***χ* ^2^_df _(*P*)^c^**

**Total**							

	1831	249 (14.5)	NA	213 (11.4)	NA	477 (25.6)	NA

**Age, y**							

18-44	824	138 (17.1)	11.9_1.98_ (<.001)	92 (11.0)	0.6_1.95_ (.53)	146 (17.4)	35.5_1.98_ (<.001)
45-64	640	96 (14.9)		82 (12.6)		192 (30.9)	
≥65	357	14 (4.5)		39 (10.3)		138 (42.6)	

**Sex**							

Male	754	81 (11.9)	6.6_1.00_ (.01)	64 (7.9)	15.8_1.00_ (<.001)	164 (21.0)	14.1_1.00_ (<.001)
Female	1077	168 (16.9)		149 (14.6)		313 (29.8)	

**Race and ethnicity**							

Non-Hispanic white	1604	190 (12.2)	16.5_2.00_ (<.001)	186 (11.6)	1.2_1.99_ (.30)	423 (26.4)	1.3_1.99_ (.27)
Hispanic or Latino	110	38 (34.0)		10 (7.0)		26 (20.7)	
Other	103	19 (18.9)		14 (12.5)		24 (20.7)	

**Education level**							

Less than high school	143	29 (23.2)	4.2_1.93_ (.02)	10 (7.0)	1.4_1.95_ (.25)	36 (23.5)	0.5_1.97_ (.63)
High school graduate/GED	534	78 (14.8)		72 (12.3)		135 (24.4)	
More than high school	1151	142 (12.9)		131 (11.6)		306 (26.5)	

**Annual household income**							

<$25,000	547	77 (14.0)	1.4_1.99_ (.24)	73 (12.8)	0.8_2.00_ (.47)	135 (23.0)	1.4_2.00_ (.24)
$25,000-$49,999	532	65 (11.9)		57 (10.1)		138 (25.5)	
≥$50,000	564	83 (15.8)		62 (11.3)		153 (27.9)	

**Family history of diabetes^d^**							

Parent or sibling with diabetes	492	150 (31.0)	106.4_1.00_ (<.001)	103 (21.1)	49.8_1.00_ (<.001)	184 (37.7)	41.1_1.00_ (<.001)
No parent or sibling with diabetes	1250	96 (8.3)		107 (7.8)		283 (21.0)	

**BMI^e^**							

Healthy weight	745	65 (9.5)	19.8_2.00_ (<.001)	68 (9.7)	8.6_2.00_ (<.001)	147 (19.7)	10.7_2.00_ (<.001)
Overweight	636	76 (12.4)		63 (9.4)		177 (26.8)	
Obese	362	92 (25.4)		70 (18.3)		129 (34.1)	

**Physical activity^f^**							

Met recommendations	912	103 (11.5)	6.4_2.00_ (.002)	108 (11.4)	2.1_1.99_ (.12)	230 (24.1)	0.5_2.00_ (.62)
Insufficient activity	639	105 (17.5)		83 (12.7)		171 (26.3)	
No activity	180	36 (20.8)		14 (6.8)		50 (26.7)	

**History of high blood pressure^g^**							

Yes	418	53 (12.7)	1.0_1.00_ (.32)	65 (15.6)	7.2_1.00_ (.007)	168 (41.0)	49.4_1.00_ (<.001)
No	1410	196 (14.9)		148 (10.3)		308 (21.5)	

**History of high cholesterol^g^**							

Yes	484	62 (14.1)	0.3_1.00_ (.57)	67 (14.1)	0.4_1.00_ (.52)	181 (39.1)	11.8_1.00_ (<.001)
No	909	112 (12.8)		116 (12.7)		250 (28.8)	

**Cigarette smoking**							

Never smoked	934	127 (14.9)	0.3_1.99_ (.76)	100 (10.5)	0.7_2.00_ (.50)	232 (24.7)	1.7_2.00_ (.19)
Former smoker	502	68 (14.2)		62 (12.0)		148 (29.2)	
Current smoker	385	53 (13.2)		51 (12.9)		96 (23.8)	

**Health care coverage**							

Yes	1515	197 (13.7)	2.7_1.00_ (.10)	184 (12.0)	2.6_1.00_ (.11)	428 (28.1)	15.9_1.00_ (<.001)
No	309	52 (18.1)		28 (8.6)		47 (15.6)	

**Personal health care provider**							

Yes	1445	193 (13.6)	1.8_1.00_ (.18)	184 (12.7)	6.3_1.00_ (.01)	430 (29.9)	30.2_1.00_ (<.001)
No	381	55 (16.8)		29 (7.5)		46 (12.9)	

**Could not seek medical care at some time in previous 12 months because of cost**							

Yes	259	50 (19.6)	4.9_1.00_ (.03)	35 (13.0)	0.6_1.00_ (.43)	55 (21.6)	1.8_1.00_ (.18)
No	1571	198 (13.5)		178 (11.1)		421 (26.1)	

**Number of risk factors^h^**							

0	446	33 (7.6)	18.1_2.99_ (<.001)	31 (6.7)	7.8_2.98_ (<.001)	52 (11.5)	26.1_2.99_ (<.001)
1	646	69 (11.7)		71 (11.4)		178 (26.5)	
2	365	80 (22.5)		59 (15.3)		127 (36.1)	
3 or 4	139	44 (31.0)		29 (21.5)		61 (44.1)	

**Age and BMI**							

≥45 years and ≥25.0 kg/m^2^	370	37 (10.2)	3.6_1.00_ (.06)	38 (10.3)	0.4_1.00_ (.52)	126 (35.4)	18.3_1.00_ (<.001)
Other	1366	195 (14.8)		163 (11.7)		326 (23.0)	

a Respondents who reported they were “very” or “somewhat” worried.

b Data are weighted percentages and number of respondents with “yes” response for each category. NA indicates not applicable.

c P <.05 indicates that diabetes risk perception, diabetes discussions with a health care professional, and diabetes testing are associated with a characteristic or risk factor based on the complex survey-design Pearson’s chi-square test but do not specify which groups are significantly different from each other. The design-based F statistic was used to calculate the chi-square value.

d Having a blood-related family member (parent or sibling) with diabetes, excluding female relatives diagnosed with diabetes only during pregnancy.

e BMI indicates body mass index. Data are based on computed BMI from self-reported height and weight ^2^ healthy weight: <25.0 kg/m2^2^, overweight: 25.0 to 29.9 kg/m^2^, obese: ≥30.0 kg/m^2^

f *Met Centers for Disease Control and Prevention (CDC) recommendations:* moderate-intensity activity during leisure time for 30 minutes or more on 5 or more days per week or vigorous activity during leisure time for 20 minutes or more on 3 or more days per week; *insufficient activity:* some physical activity during leisure time but not enough to meet CDC recommendations; *no activity:* less than 10 minutes of moderate-intensity physical activity during leisure time in a usual week.

g Ever been diagnosed with high blood pressure or high cholesterol by doctor, nurse, or other health professional.

h Risk factors include family history of diabetes, obesity, ages 45 years and older, and inactivity.

**Table 3:** Independent Associations Among Selected Characteristics of Oregon Adults Without Diabetes Who Were Worried About Developing Diabetes in the Next 10 Years, 2003 Oregon Behavioral Risk Factor Surveillance System^a^

**Characteristics**	**Bivariate Odds Ratio (95% CI)^b^**	**Adjusted Odds Ratio (95% CI)^b^ (N = 1582)**

**Age, y**		

18-44	1.0 (ref)	1.0 (ref)
45-64	0.85 (0.61-1.18)	0.68 (0.46-1.01)
≥65	0.23 (0.12-0.42)	0.21 (0.11-0.41)

**Sex**		

Male	1.0 (ref)	1.0 (ref)
Female	1.51 (1.10-2.07)	1.56 (1.07-2.29)

**Race and ethnicity**		

Non-Hispanic white	1.0 (ref)	1.0 (ref)
Hispanic or Latino	3.70 (2.29-5.97)	2.58 (1.28-5.18)
Other	1.68 (0.90-3.13)	0.86 (0.35-2.11)

**Education level**		

Less than high school	1.0 (ref)	1.0 (ref)
High school graduate/GED	0.57 (0.33-1.00)	1.09 (0.43-2.72)
More than high school	0.49 (0.29-0.83)	1.37 (0.53-3.50)

**Family history of diabetes**		

No parent or sibling with diabetes	1.0 (ref)	1.0 (ref)
Parent or sibling with diabetes	4.96 (3.59-6.85)	4.68 (3.27-6.71)

**BMI^c^**		

Healthy weight	1.0 (ref)	1.0 (ref)
Overweight	1.36 (0.91-2.02)	1.29 (0.82-2.04)
Obese	3.25 (2.19-4.82)	2.80 (1.78-4.40)

**Physical activity^d^**		

Met recommendations	1.0 (ref)	1.0 (ref)
Insufficient activity	1.64 (1.17-2.29)	1.56 (1.06-2.32)
No activity	2.03 (1.26-3.25)	1.79 (0.94-3.39)

**Could not seek medical care at some time in previous 12 months because of cost**		

No	1.0 (ref)	1.0 (ref)
Yes	1.56 (1.05-2.32)	1.34 (0.81-2.24)

**Number of risk factors^e^**		

0	1.0 (ref)	NA
1	1.60 (0.99-2.60)	NA
2	3.51 (2.18-5.64)	NA
3 or 4	5.43 (3.08-9.55)	NA

a Respondents who reported they were “very” or “somewhat” worried.

b CI indicates confidence interval; ref, reference level for characteristic; and NA, not applicable.

c BMI indicates body mass index. Data are based on computed BMI from self-reported height and weight — healthy weight: <25.0 kg/m^2^, overweight: 25.0 to 29.9 kg/m^2^, obese: ≥30.0 kg/m^2^.

d *Met Centers for Disease Control and Prevention (CDC) recommendations:* moderate-intensity activity during leisure time for 30 minutes or more on 5 or more days per week or vigorous activity during leisure time for 20 minutes or more on 3 or more days per week; *insufficient activity:* some physical activity during leisure time but not enough to meet CDC recommendations; *no activity:* less than 10 minutes of moderate-intensity physical activity during leisure time in a usual week.

e Risk factors include family history of diabetes, obesity, ages 45 years and older, and inactivity.

**Table 4:** Independent Associations Among Selected Characteristics of Oregon Adults Without Diabetes Who Had Talked With a Health Care Professional About Diabetes in the Previous Year, 2003 Oregon Behavioral Risk Factor Surveillance System

**Characteristics**	**Bivariate Odds Ratio (95% CI)^a^**	**Adjusted Odds Ratio (95% CI)^a^ (N = 1655)**

**Sex**		

Male	1.0 (ref)	1.0 (ref)
Female	1.99 (1.41-2.81)	1.80 (1.23-2.63)

**Family history of diabetes**		

No parent or sibling with diabetes	1.0 (ref)	1.0 (ref)
Parent or sibling with diabetes	3.16 (2.27-4.41)	2.89 (2.03-4.10)

**BMI^b^**		

Healthy weight	1.0 (ref)	1.0 (ref)
Overweight	0.97 (0.64-1.46)	0.91 (0.59-1.39)
Obese	2.09 (1.39-3.13)	1.62 (1.06-2.47)

**History of high blood pressure^c^**				

No	1.0 (ref)	1.0 (ref)
Yes	1.62 (1.14-2.31)	1.34 (0.91-1.96)

**Have personal health care provider**				

No	1.0 (ref)	1.0 (ref)
Yes	1.80 (1.13-2.86)	1.50 (0.92-2.47)

**Number of risk factors^d^**		

0	1.0 (ref)	NA
1	1.81 (1.10-2.98)	NA
2	2.52 (1.50-4.24)	NA
3 or 4	3.83 (2.07-7.12)	NA

a CI indicates confidence interval; ref, reference level for characteristic; and NA, not applicable.

b BMI indicates body mass index. Data are based on computed BMI from self-reported height and weight — healthy weight: <25.0 kg/m^2^, overweight: 25.0 to 29.9 kg/m^2^, obese: ≥30.0 kg/m^2^

c Ever been diagnosed with high blood pressure by doctor, nurse, or other health professional.

d Risk factors include family history of diabetes, obesity, ages 45 years and older, and inactivity.

**Table 5:** Independent Associations Among Selected Characteristics of Oregon Adults Without Diabetes Who Were Tested for Diabetes by a Health Care Provider in the Previous Year, 2003 Oregon Behavioral Risk Factor Surveillance System

**Characteristics**	**Bivariate Odds Ratio (95% CI)^a^**	**Adjusted Odds Ratio (95% CI)^a^ (N = 1217)**

**Age, y**		

18-44	1.0 (ref)	1.0 (ref)
45-64	2.12 (1.61-2.80)	1.17 (0.83-1.66)
≥65	3.52 (2.58-4.80)	1.86 (1.25-2.76)

**Sex**		

Male	1.0 (ref)	1.0 (ref)
Female	1.59 (1.25-2.04)	1.30 (0.97-1.74)

**Family history of diabetes**		

No parent or sibling with diabetes	1.0 (ref)	1.0 (ref)
Parent or sibling with diabetes	2.28 (1.76-2.94)	2.03 (1.49-2.76)

**BMI^b^**		

Healthy weight	1.0 (ref)	1.0 (ref)
Overweight	1.49 (1.12-1.98)	1.15 (0.83-1.59)
Obese	2.10 (1.52-2.90)	1.47 (0.99-2.19)

**History of high blood pressure**		

No	1.0 (ref)	1.0 (ref)
Yes	2.54 (1.95-3.31)	1.51 (1.09-2.11)

**History of high cholesterol**		

No	1.0 (ref)	1.0 (ref)
Yes	1.59 (1.22-2.08)	1.14 (0.84-1.55)

**Have health care coverage**		

No	1.0 (ref)	1.0 (ref)
Yes	2.12 (1.46-3.09)	1.53 (0.92-2.53)

**Have personal health care provider**		

No	1.0 (ref)	1.0 (ref)
Yes	2.88 (1.95-4.26)	1.45 (0.87-2.43)

**Number of risk factors^c^**		

0	1.0 (ref)	NA
1	2.78 (1.93-4.01)	NA
2	4.35 (2.92-6.48)	NA
3 or 4	6.07 (3.67-10.01)	NA

**Age and BMI**		

≥45 years and BMI ≥25.0 kg/m^2^	1.84 (1.39-2.43)	NA

a CI indicates confidence interval; ref, reference level for characteristic; and NA, not applicable.

b BMI indicates body mass index. Data are based on computed BMI from self-reported height and weight — healthy weight: <25.0 kg/m2, overweight: 25.0 to 29.9 kg/m2, obese: ≥30 kg/m2

c Risk factors include family history of diabetes, obesity, ages 45 years and older, and inactivity.
